# The effect of 12 weeks of aerobic, resistance or combination exercise training on cardiovascular risk factors in the overweight and obese in a randomized trial

**DOI:** 10.1186/1471-2458-12-704

**Published:** 2012-08-28

**Authors:** Suleen S Ho, Satvinder S Dhaliwal, Andrew P Hills, Sebely Pal

**Affiliations:** 1 School of Public Health; Curtin Health Innovation Research Institute, Curtin University of Technology, GPO Box U1987, Perth, Western Australia, Australia, 6845; 2 Mater Mother’s Hospital, Mater Medical Research Institute. Conjoint appointment with Griffith Health Institute, Griffith University, Brisbane, Australia

**Keywords:** Obesity, Overweight, Cardiovascular risk factors, Exercise training

## Abstract

**Background:**

Evidence suggests that exercise training improves CVD risk factors. However, it is unclear whether health benefits are limited to aerobic training or if other exercise modalities such as resistance training or a combination are as effective or more effective in the overweight and obese. The aim of this study is to investigate whether 12 weeks of moderate-intensity aerobic, resistance, or combined exercise training would induce and sustain improvements in cardiovascular risk profile, weight and fat loss in overweight and obese adults compared to no exercise.

**Methods:**

Twelve-week randomized parallel design examining the effects of different exercise regimes on fasting measures of lipids, glucose and insulin and changes in body weight, fat mass and dietary intake. Participants were randomized to either: Group 1 (Control, n = 16); Group 2 (Aerobic, n = 15); Group 3 (Resistance, n = 16); Group 4 (Combination, n = 17). Data was analysed using General Linear Model to assess the effects of the groups after adjusting for baseline values. Within-group data was analyzed with the paired *t*-test and between-group effects using post hoc comparisons.

**Results:**

Significant improvements in body weight (−1.6%, p = 0.044) for the Combination group compared to Control and Resistance groups and total body fat compared to Control (−4.4%, p = 0.003) and Resistance (−3%, p = 0.041). Significant improvements in body fat percentage (−2.6%, p = 0.008), abdominal fat percentage (−2.8%, p = 0.034) and cardio-respiratory fitness (13.3%, p = 0.006) were seen in the Combination group compared to Control. Levels of ApoB48 were 32% lower in the Resistance group compared to Control (p = 0.04).

**Conclusion:**

A 12-week training program comprising of resistance or combination exercise, at moderate-intensity for 30 min, five days/week resulted in improvements in the cardiovascular risk profile in overweight and obese participants compared to no exercise. From our observations, combination exercise gave greater benefits for weight loss, fat loss and cardio-respiratory fitness than aerobic and resistance training modalities. Therefore, combination exercise training should be recommended for overweight and obese adults in National Physical Activity Guidelines.

This clinical trial was registered with the Australian New Zealand Clinical Trials Registry (ANZCTR), registration number: ACTRN12609000684224.

## Background

Physical activity is a major modifiable determinant of chronic disease [1]. The Australian National Physical Activity Guidelines for Adults recommend that for good health, adults should “put together at least 30 min of moderate-intensity physical activity on most, preferably all, days [2].” However, it is not known if this recommendation is adequate for improvement in cardiovascular disease (CVD) risk factors in overweight and obese individuals [3]. Despite the acknowledged role of 30 minutes of daily physical activity on general health improvements in an otherwise healthy but sedentary population, less is known of the adequacy of this level of exercise for health improvements in those who are overweight or obese. In addition, as many people have difficulty finding time to exercise, it is important to better understand which mode(s) of exercise is the most effective.

Numerous studies have investigated the effects of exercise training, demonstrating significant improvements to CVD risk factors after aerobic exercise training [4,5]. However, it is unclear whether health benefits are limited to aerobic training or if other exercise modalities such as resistance training or a combination are as effective or more effective in the overweight and obese.

Sigal et al. [6] investigated the effects of aerobic, resistance and combined aerobic and resistance training in adults with Type 2 diabetes. However, the combined intervention used both aerobic (45-min walking/cycling) plus resistance training (2–3 sets of 7 exercises with 7–9 repetitions), that is, participants completed a double dose of exercise. They observed significant decreases in body weight, body mass index (BMI) and abdominal subcutaneous fat in the aerobic and resistance groups compared to control.

Davidson et al. [7] also examined different exercise modalities in older adults. Calorie intake was strictly controlled and results were due solely to the energy deficit of the exercise interventions. They observed significant improvements to total, abdominal and visceral fat and cardio-respiratory fitness in the aerobic and combination exercise groups. It was concluded that the combination of the resistance and aerobic exercise was the optimal exercise strategy for improvements to insulin resistance and functional limitations [7].

A recent study by Church et al. [8] compared equivalent time durations (140 min/week) of aerobic, resistance and combination exercise. They observed significant improvements in HbA_1c_ and maximum oxygen consumption in the combination group compared to control as well as decreases in weight and fat mass in the resistance and combination groups compared to control.

Research comparing the effect of resistance and aerobic training on CVD risk factors is limited and few studies have compared aerobic, resistance and a combination of this training. Given the increasing burden of chronic disease, more research is needed to better understand the effect of different exercise modalities on these risk factors. Australian Physical Activity Guidelines promote 30-min of moderate-intensity exercise on most days of the week; therefore the aim of this study was to investigate whether a 12-week training program with aerobic, resistance, or combined exercise at the same intensity and duration would improve the cardiovascular risk profile in overweight and obese adults compared to no exercise.

## Methods

### Participants

**Figure 1:**
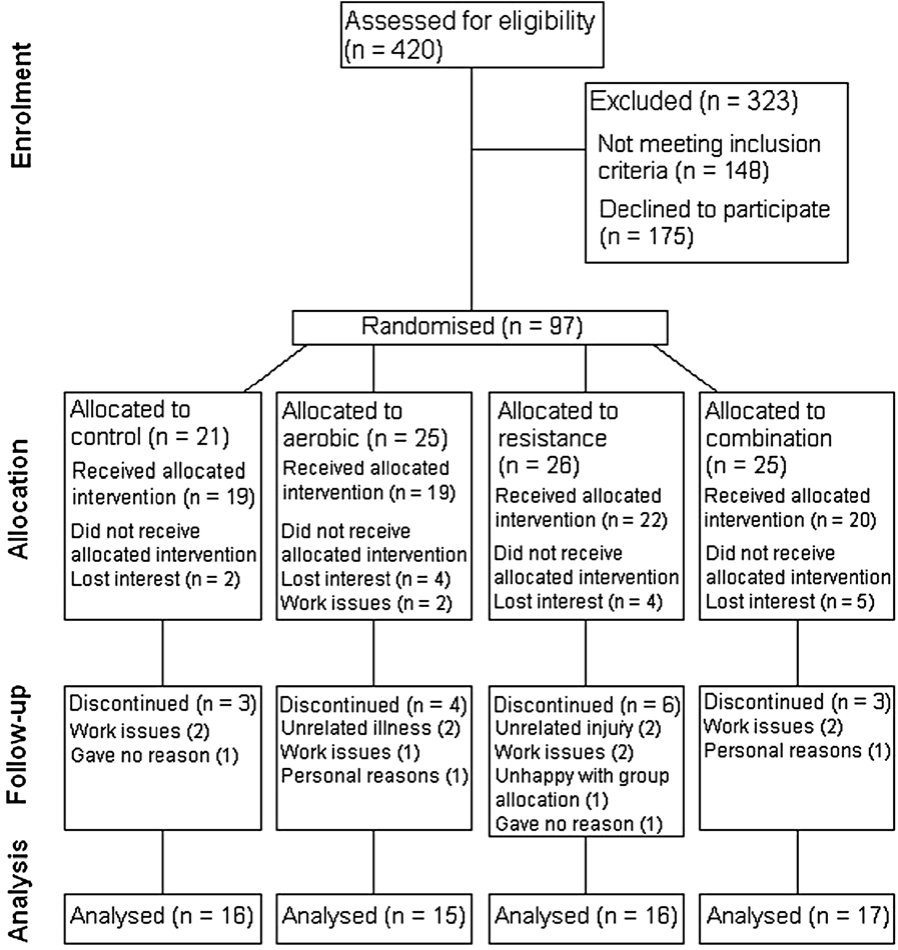
Participant flow chart.

Ninety-seven overweight or obese men (n = 16) and women (n = 81) (BMI >25 kg/m^2^ or waist circumference >80 cm for women and 90 cm for men), aged 40 to 66 y were recruited from the general population from April 2006 - April 2007 (Figure 1). Participants were required to be sedentary or relatively inactive, participating in less than 1 h of moderate-intensity physical activity per week over the last 3 months. Participants were recruited from an existing database of volunteers from previous studies that concluded at least 6 months prior and from the community using local newspapers and radio. Interested individuals were screened via telephone. Exclusion criteria included diabetes mellitus, pre-existing heart conditions, lipid lowering medication, beta-blockers, pregnant or lactating women, smokers, gastrointestinal tract surgery, major illness (acute or chronic) including any that would limit the ability to perform the necessary exercises. Study procedures were approved by the Curtin University Ethics Committee (HR 166/2004) and participants gave written informed consent.

### Study design

This 12-week study was a randomized parallel design examining the effects of different exercise regimes on fasting measures and changes in body weight, fat mass and dietary intake. Participants were randomized to four different groups as they were recruited by the researcher (using a randomization sequence [9]). Group 1 (Control) were not given any exercise intervention (placebo dietary supplement only). This was to ensure participants did not know they were in the control group and followed study procedures similarly to those in the intervention groups. Control participants were asked to take a teaspoon of supplement in a glass of water once/day which contained approximately 2 grams breadcrumbs and 0.1 grams “Equal” artificial sweetener. Group 2 (Aerobic) performed 30-min of aerobic exercise, 5 days/week, consisting of treadmill walking. Group 3 (Resistance) performed 30-min of resistance exercise, 5 days/week using weight resistance machines. Group 4 (Combination) performed 15-min of aerobic and 15-min of resistance exercise 5 days/week. Three assessment visits were conducted in clinical rooms at Curtin University for blood samples, body composition and other measurements. All participants were requested to keep food intake and physical activity the same as before the study, except for those in the exercise groups, who were instructed to do additional exercise as per their program. Participants completed baseline measurements over one week before attending an initiation session at the Curtin Fitness Centre where their exercise program was demonstrated by Centre staff.

### Exercise interventions

The exercise interventions were either 30-min aerobic exercise on a treadmill at 60% heart rate reserve (HRR) ±10 beats/min, with HRR estimated using the Karvonen equation (220-age-resting heart rate) [10]; 30-min of resistance exercise (four sets of 8–12 repetitions at 10-RM for leg press, leg curl, leg extension, bench press and rear deltoid row, with each set completed in approximately 30-sec with 1-min rest) with the 10-RM level determined during the initial session by Fitness Centre staff; or a combination of 15-min aerobic exercise and 15-min of resistance exercise (two sets of each exercise). 10-RM would be approximately 75% of 1-RM [11]. Starting workload levels for each piece of equipment were tested by participants and if more than 10 repetitions were achieved, the weight was increased and after a short rest participants tried again. Likewise, if less than 8 repetitions were achieved, the weight was decreased and after a short rest participants tried again. Participants began by exercising three days/week for two weeks before exercising at recommended levels for five days/week thereafter. Participants reported to the Curtin Fitness Centre three days/week to complete the required exercise and either exercised at home the other two days or at the Centre. If exercises were completed at home, dumbbells (adjustable weight 1.5-10.5 kg) were provided for resistance exercises (three sets of 10 repetitions for biceps curls, lunges, dumbbell raise, calf lift, triceps extension for Resistance group while the Combination group did 2 sets of biceps curls and lunges and 1 set for dumbbell raise, calf lift and triceps extension; back extension, push ups and sit ups exercises were also included). Treadmills were equipped with heart rate sensors and participants were instructed to increase weight loads by 2.5 kg increments when they could complete more than 12 repetitions. Participants kept food diaries to monitor dietary intake and were given instructions during the briefing session. Data was analysed with Foodworks Professional 2007, Xyris Software, Australia.

### Measurement of biochemical markers

Participants reported for fasting blood samples at baseline and at the completion of 8 and 12 weeks of training. Serum triglyceride (TG) and total cholesterol were measured by enzymatic colorimetric kits (TRACE Scientific LTD, Melbourne, Australia). High density lipoprotein (HDL)-cholesterol was determined after precipitation of apoB-containing lipoproteins with phosphotungstic acid and MgCl_2_[12]. Low density lipoprotein (LDL)-cholesterol was determined using a modified version of the Friedewald equation [13]. Non-esterified fatty acid was determined in serum using the WAKO NEFA C Kit (Wako Pure Chemicals Industries, Osaka, Japan) according to instructions, but scaled down 1:10 to use on microtiter plates. Plasma glucose was measured using Randox glucose GOD-PAP kits (Randox, Antrim, United Kingdom). Plasma insulin was measured by a solid phase Enzyme Amplified Sensitivity Immunoassay (INS-EASIA kit, BioSource, Belgium). Homeostasis model assessment of insulin resistance (HOMA2-IR) was calculated from fasting glucose and insulin concentrations [14].

### Anthropometric measures

Anthropometric measures were completed and BMI calculated. Weight was measured using electronic scales (Tanita Corporation, Tokyo, Japan). Waist circumference was measured at the mid-point between the bottom of the rib cage and the iliac crest and hip circumference was measured at the widest point at the hip. Abdominal and total body fat was measured by DXA (dual-energy X-ray absorptiometry) at baseline and 12 weeks (GE-Lunar Prodigy DXA scanner, GE Healthcare, Chalfont St. Giles, UK). The analysis program automatically defines the abdominal fat region with a rectangle from the upper edge of the second lumbar vertebra extended to the lower edge of the fourth lumbar vertebra.

### Apolipoprotein B48 determination

Plasma samples and purified apoB48 standards (previously prepared according to Zilversmit and Shea [15]) were separated by SDS-PAGE using precast NuPAGE 3-8% gradient gels in a Novex Mini-Cell (Novex Instruments, CA, USA) at 150 V for 80 min as described previously [16]. Separated proteins were electro-transferred at 30 V for 90 min onto 0.45 μm polyvinylidine diflouride (PVDF) membrane. Membranes were blocked overnight at 4 °C in TBST (tris-buffered saline with Tween-20) (10 mmol/L Tris–HCl buffer, pH 7.4, containing 154 mmol/L NaCl) and 10% (w/v) skim milk powder. After washing in TBST, membranes were incubated with 5 μg/ml rabbit anti-human apoB antibody in TBST for 60 min. After washing in TBST, membranes were incubated with 0.5 μg/ml donkey anti-rabbit IgG linked to horseradish peroxidase in TBST for 45 min. After washing in TBST, membranes were incubated with enhanced chemiluminescence substrate solution for detection of horseradish peroxidase and exposed to hyper-film ECL. The film was developed using an AGFA CP1000 automatic film processor, and apoB48 determined by densitometric scanning using the ScanMaker 9800XL (Microtek International, USA) scanner and Scion Image program (Scion Inc).

### Post-absorptive energy expenditure

Resting energy expenditure (REE) and respiratory quotient (RQ) were measured in the fasted state for 30 min at baseline and week 12 by indirect open-circuit calorimetry using a ventilator canopy attached to the Deltatrac II Metabolic Monitor (GE Healthcare). All metabolic measurements were conducted using proven methodology [17] under standardized conditions used in our laboratory. Gas calibration was conducted once a day, prior to any measurements. Before measurements were taken, participants rested in a supine position for 10–15 min.

### Cardio-respiratory fitness assessment

Cardio-respiratory fitness (CRF) was assessed using the Astrand-Rhyming Submaximal Cycle Ergometer Test [18] (Monark Exercise AB, Sweden). This was a single-stage, 6-min test to estimate maximal oxygen consumption (VO_2_max) from prediction tables of maximal oxygen consumption at baseline and week 12. A submaximal protocol was chosen as this method is less stressful and safer for overweight or obese individuals who are also sedentary.

### Statistical analysis

Sample size calculations were based on a minimum predicted 15% change in fasting triglyceride and total cholesterol levels between the intervention groups, with an expected standard deviation of 15%. A sample size of 16 participants per group was predicted to provide sufficient power (80%) to detect significant changes at the 5% significance level. However, we aimed to recruit 20 participants per group (a total of 80) to accommodate for a 20% attrition rate and elimination due to non-compliance.

Data was assessed for normality to ensure that the assumptions of the analysis were met. The data for fasting total cholesterol, LDL cholesterol, HDL cholesterol, triglyceride, glucose, insulin and anthropometric measures was analysed using General Linear Model to assess the effects of the groups after adjusting for group and baseline values. Within-group data was analyzed with the paired *t*-test.

When significant between-group effects were present, post hoc comparisons were made using the LSD method. Statistical analysis was carried out using SPSS 14.0 for Windows (SPSS Inc., Chicago, IL, USA).

## Results

Participant characteristics at baseline can be seen in Table 1.

**Table 1:** Participant characteristics at baseline

**Characteristic**	**Control (n = 16)**	**Aerobic (n = 15)**	**Resistance (n = 16)**	**Combination (n = 17)**
Male/Female	1/15	3/12	3/13	3/14
Age (years)	52 ± 1.8	55 ± 1.2	52 ± 1.1	53 ± 1.3
	(40 – 66)	(44 – 62)	(43 – 59)	(43 – 64)
Weight (kg)	85.1 ± 4.2	91.9 ± 4.1	89.3 ± 4.5	90 ± 4
	(64.8 – 123)	(65.9 – 124.1)	(71.9 – 127.5)	(62.2 – 122.3)
Body Mass Index (kg/m^2^)	32.4 ± 1.4	32.7 ± 1.3	33 ± 1.3	33.3 ± 1.2
	(26 – 48)	(25 – 45.6)	(25.8 – 44.6)	(23.4 – 40.2)
Body Fat % (DXA)	46.5 ± 1.7	44.6 ± 1.9	43.7 ± 1.3	45.8 ± 1.6
	(35.9 – 59.9)	(30.7 – 52.5)	(34.6 – 52.2)	(28.8 – 55.5)
Waist Circumference (cm)	100.3 ± 3.6	103.7 ± 2.6	104 ± 3.2	102.2 ± 3.2
	(80 – 131)	(82 – 118)	(83.5 – 135.5)	(81.5 – 124.5)
Waist: Hip Ratio	0.85 ± 0.02	0.87 ± 0.02	0.88 ± 0.02	0.86 ± 0.02
	(0.75 – 1.01)	(0.76 – 1)	(0.78 – 1.03)	(0.74 – 1.02)
Fasting Triglyceride (mmol/L)	1.25 ± 0.17	1.36 ± 0.19	1.27 ± 0.12	1.1 ± 0.1
	(0.48 – 2.6)	(0.62 – 3.01)	(0.51 – 2.14)	(0.48 – 1.91)
Fasting Total Cholesterol (mmol/L)	5.51 ± 0.29	5.83 ± 0.32	5.49 ± 0.38	5.71 ± 0.31
	(2.59 – 7.12)	(3.58 – 7.87)	(2.92 – 8.95)	(3.74 – 9.2)
Fasting HDL Cholesterol (mmol/L)	1.42 ± 0.11	1.38 ± 0.09	1.34 ± 0.08	1.43 ± 0.11
	(0.9 – 2.28)	(0.91 – 1.99)	(0.91 – 2.19)	(0.71 – 2.08)
Fasting LDL Cholesterol (mmol/L)	3.51 ± 0.26	3.89 ± 0.3	3.56 ± 0.33	3.78 ± 0.27
	(1.42 – 5.26)	(2.08 – 6.06)	(1.58 – 7.02)	(2.4 – 6.88)
Fasting Glucose (mmol/L)	5.35 ± 0.13	5.68 ± 0.17	5.81 ± 0.46	5.38 ± 0.13
	(4.55 – 6.22)	(4.39 – 7.11)	(4.91 – 7.17)	(4.2 – 6.46)
HOMA2-IR	1.92 ± 0.28	1.72 ± 0.52	1.86 ± 0.18	1.86 ± 0.13
	(1 – 4.7)	(0.9 – 2.5)	(0.7 – 3.5)	(1.2 – 2.8)
NEFA (mmol/L)	0.51 ± 0.03	0.5 ± 0.04	0.49 ± 0.04	0.46 ± 0.03
	(0.3 – 0.79)	(0.17 – 0.87)	(0.18 – 0.94)	(0.29 – 0.7)

Values are mean ± SEM and (range) for n = 64 participants at baseline. BIA: bioelectrical impedance analysis. DXA: dual-energy x-ray absorptiometry. HOMA2-IR: homeostasis model assessment of insulin resistance. NEFA: non-esterified fatty acid.

The average daily energy intake from 3-day food diaries at baseline, week 8 and week 12 can be seen in Table 2. When comparing within-group changes, the Aerobic and Resistance groups had significantly lower daily energy intake (EI) at week 12 compared to baseline (18%, *p* = 0.041 and 11%, *p* = 0.039 decrease, respectively). However, there were no significant differences in total energy intake between groups at week 12. Participants attended 67-74% of exercise sessions, with no significant difference between intervention groups.

**Table 2:** Changes in nutritional variables

	**Baseline**	**Week 8**	**Week 12**
**Energy Intake (EI)**			
Control	7085.1 ± 550.8	7535.6 ± 1197.8	6723.2 ± 585.9
Aerobic	8741.6 ± 1052.7	7471.7 ± 706.7	7179.4 ± 502.3*
Resistance	7776.6 ± 418.0	7234.5 ± 478.0	6937.1 ± 471.6*
Combination	7675.3 ± 324.8	7726.5 ± 428.6	6920.2 ± 402.7
**Carbohydrate % of EI**			
Control	41.8 ± 3.0	42.1 ± 1.8	40.7 ± 2.5
Aerobic	42.5 ± 3.1	44.4 ± 2.6	41.2 ± 2.3
Resistance	40.2 ± 1.6	41.7 ± 1.9	39.4 ± 2.0
Combination	44.2 ± 1.9	44.8 ± 1.9	43.4 ± 1.9
**Protein % of EI**			
Control	18.9 ± 1.5	20.1 ± 1.1	18.5 ± 1.2
Aerobic	18.3 ± 1.3	19.7 ± 1.4	20.8 ± 1.4*
Resistance	19.8 ± 1.1	19.0 ± 0.7	20.2 ± 1.0
Combination	19.1 ± 0.9	18.5 ± 1.0	18.4 ± 1.1
**Fat % of EI**			
Control	33.5 ± 2.2	34.0 ± 1.5	37.1 ± 1.8
Aerobic	35.2 ± 2.6	33.1 ± 1.9	35.1 ± 1.8
Resistance	36.0 ± 1.2	34.8 ± 1.8	36.4 ± 1.4
Combination	33.9 ± 1.7	35.7 ± 1.7	35.7 ± 1.4
**SFA % of Fat**			
Control	41.6 ± 2.1	40.2 ± 1.5	43.6 ± 2.1
Aerobic	41.6 ± 2.3	41.8 ± 1.6	42.5 ± 1.9
Resistance	40.4 ± 1.8	43.3 ± 1.8	42.6 ± 1.0
Combination	45.4 ± 1.6	45.5 ± 1.6	42.8 ± 3.0
**MUFA % of Fat**			
Control	40.6 ± 1.4	41.4 ± 1.1	39.6 ± 1.3
Aerobic	41.3 ± 1.5	39.6 ± 0.8	38.8 ± 1.2
Resistance	39.9 ± 0.9	40.3 ± 1.0	40.8 ± 1.2
Combination	38.9 ± 1.0	39.5 ± 1.0	39.5 ± 1.1
**PUFA % of Fat**			
Control	17.7 ± 1.5	18.4 ± 1.1	16.9 ± 1.4
Aerobic	17.1 ± 1.2	18.4 ± 1.6	18.6 ± 1.8
Resistance	19.6 ± 1.5	16.6 ± 1.2	16.8 ± 1.1
Combination	15.9 ± 1.0	15.2 ± 0.8	17.5 ± 2.4

Values are daily mean ± SEM (n = 53) of the nutritional data recorded in 3-day food diaries at baseline, week 8 and week 12. Statistical significance from baseline is indicated by * p < 0.05. SFA: saturated fatty acid. MUFA: monounsaturated fatty acid. PUFA: polyunsaturated fatty acid.

**Figure 2:**
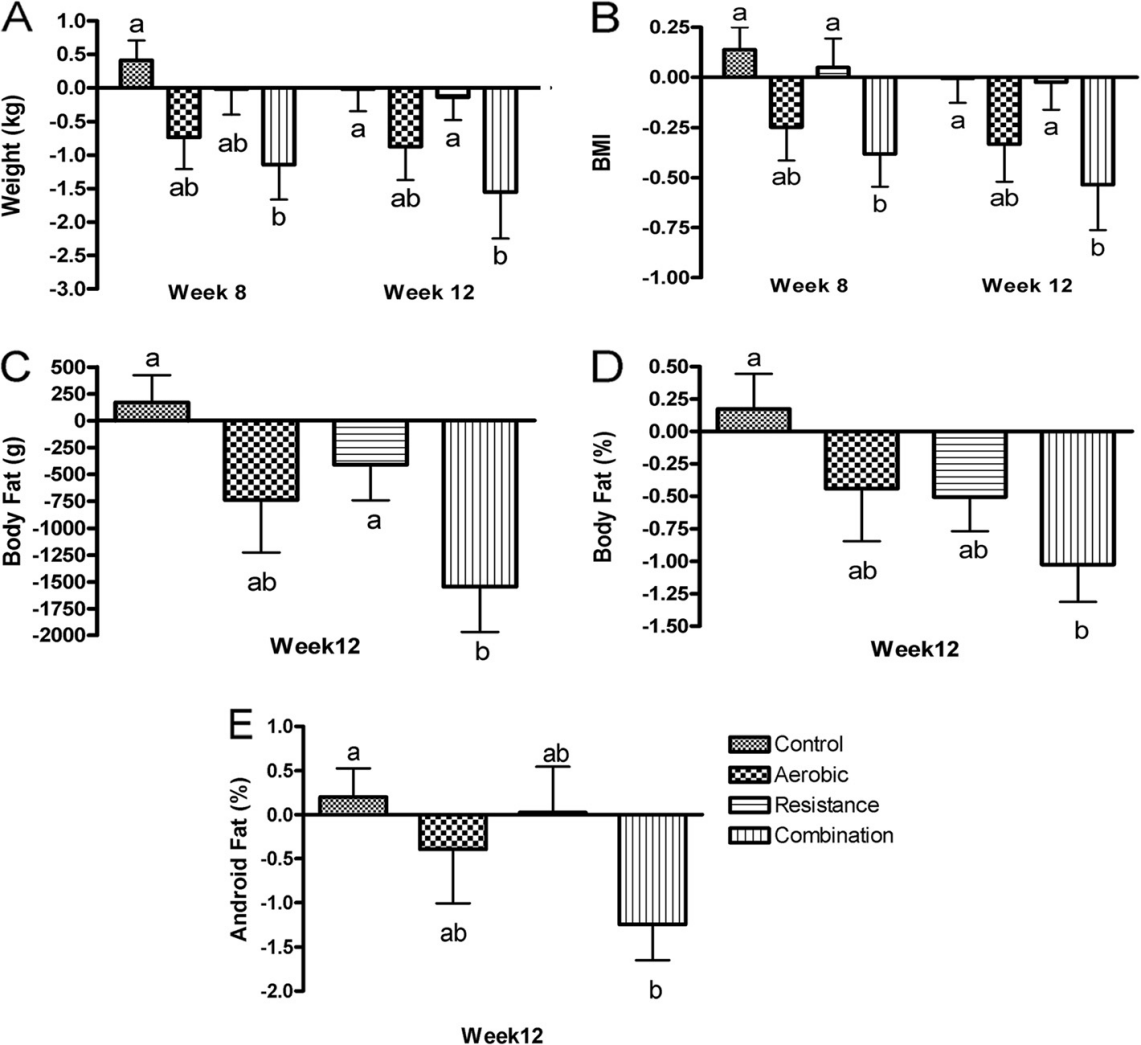
**Comparison of changes from baseline between groups for body weight (A), BMI (B), body fat (C), body fat % (D) and android fat % (E).** Body fat was measured by DXA. Data represents mean ± SEM. Statistical differences between groups indicated by different superscripts, *p* < 0.05.

In the Combination group, change in body weight was significantly lower compared to Control at week 8 (−1.6%, *p* = 0.018) and week 12 (−1.6%, *p* = 0.044) and also Resistance group at week 12 (−1.6%, *p* = 0.044) (Figure 2A). Change in BMI was significantly lower in the Combination group compared to Control (−1.6%, *p* = 0.016) and Resistance (−1.3%, *p* = 0.042) at week 8 and Control (−1.6%, *p* = 0.040) and Resistance exercise (−1.6%, *p* = 0.042) at week 12 (Figure 2B). Change in total body fat in the Combination group was significantly lower compared to Control (−4.4%, *p* = 0.003) and Resistance (−3%, *p* = 0.041) groups at week 12 (Figure 2C). This was also the case for change in fat percentage, Combination vs. Control (−2.6%, *p* = 0.008) (Figure 2D) and for change in android fat percentage (−2.8%, *p* = 0.034) (Figure 2E). These results are also summarised in Table 3.

**Table 3:** Changes in body measurements and cardio-respiratory fitness

**Weight (kg)**	**Baseline**	**Week 8**	**Week 12**
Control	85.1 ± 4.2	85.5 ± 4.3	85.1 ± 4.3
Aerobic	91.9 ± 4.1	91.1 ± 4.1	91.0 ± 4.0
Resistance	89.3 ± 4.5	89.3 ± 4.3	89.2 ± 4.4
Combination	90.0 ± 4.0	88.8 ± 3.6*	88.4 ± 3.6*
**BMI**			
Control	32.4 ± 1.4	32.5 ± 1.5	32.4 ± 1.5
Aerobic	32.7 ± 1.3	32.5 ± 1.3	32.4 ± 1.2
Resistance	33.0 ± 1.3	33.1 ± 1.3	33.0 ± 1.3
Combination	33.3 ±1.2	33.0 ± 1.1*	32.8 ± 1.1*
**Fat (kg)**			
Control	37.1 ± 2.3		37.3 ± 2.4
Aerobic	39.3 ± 2.5		38.6 ± 2.5
Resistance	37.6 ± 2.3		37.2 ± 2.4
Combination	39.3 ± 1.8		37.7 ± 1.8*
**BF%**			
Control	46.5 ± 1.7		46.7 ± 1.8
Aerobic	44.6 ± 1.9		44.1 ± 1.8
Resistance	43.7 ± 1.3		43.2 ± 1.4
Combination	45.8 ± 1.6		44.8 ± 1.8*
**Android Fat %**			
Control	51.5 ± 1.8		51.7 ± 1.8
Aerobic	50.7 ± 1.6		50.4 ± 1.9
Resistance	49.2 ± 1.3		49.3 ± 1.3
Combination	52.2 ± 1.4		50.9 ± 1.4*
**Waist (cm)**			
Control	100.3 ± 3.6	98.3 ± 3.7*	99.1 ±3.6
Aerobic	103.7 ± 2.6	102.5 ± 2.6	101.6 ± 2.9*
Resistance	104.0 ± 3.2	102.4 ± 3.2	101.4 ± 3.3*
Combination	102.2 ± 3.2	100.5 ± 2.8	99.6 ± 3.0*
**VO**_**2**_**max (mL/kg/min)**			
Control	27.2 ± 1.4		24.9 ± 0.8
Aerobic	24.8 ± 1.1		26.2 ± 0.9
Resistance	24.8 ± 1.8		26.8 ± 0.8
Combination	26.5 ± 1.3		28.2 ± 0.8*

Values are mean ± SEM (n=64) at baseline, week 8 and week 12. Statistical significance from baseline is indicated by * p < 0.05.

Table 4 summarises the results for biochemical markers and energy expenditure. Levels of HDL were significantly greater in the Resistance group compared to Aerobic exercise at weeks 8 and 12. Resistance HDL was also significantly higher than Control at week 12. In the Resistance group, apoB48 levels were significantly lower than in Controls at week 12, 32% (*p* = 0.04). In the Resistance group, RQ was significantly lower compared to Control (−4.5%, *p* = 0.037), Aerobic (−4.9%, *p* = 0.027) and Combination (−4.9%, *p* = 0.022).

**Table 4:** Changes in fasting blood measurements, resting energy expenditure and respiratory quotient

	**Baseline**	**Week 8**	**Week 12**
**TG (mmol/L)**			
Control	1.25 ± 0.17	1.37 ± 0.19	1.48 ± 0.23
Aerobic	1.36 ± 0.19	1.29 ± 0.11	1.40 ± 0.16
Resistance	1.27 ± 0.12	1.21 ± 0.16	1.38 ± 0.18
Combination	1.10 ± 0.10	1.08 ± 0.10	1.36 ± 0.17*
**TC (mmol/L)**			
Control	5.51 ± 0.29	5.88 ± 0.32	5.49 ± 0.28^a^
Aerobic	5.83 ± 0.32	5.72 ± 0.29	5.56 ± 0.37^a^
Resistance	5.49 ± 0.38	5.76 ± 0.36	6.15 ± 0.44* ^b^
Combination	5.71 ± 0.31	5.74 ± 0.27	5.75 ± 0.26^ab^
**HDL (mmol/L)**			
Control	1.42 ± 0.11	1.45 ± 0.11^ab^	1.35 ± 0.10^a^
Aerobic	1.38 ± 0.09	1.31 ± 0.08^a^	1.28 ± 0.07* ^a^
Resistance	1.34 ± 0.08	1.44 ± 0.08^b^	1.44 ± 0.08* ^b^
Combination	1.43 ± 0.11	1.44 ± 0.10^ab^	1.41 ± 0.11^ab^
**LDL (mmol/L)**			
Control	3.51 ± 0.26	3.79 ± 0.31	3.47 ± 0.25^a^
Aerobic	3.89 ± 0.30	3.82 ± 0.27	3.64 ± 0.33^a^
Resistance	3.56 ± 0.33	3.76 ± 0.31	4.08 ± 0.36* ^b^
Combination	3.78 ± 0.27	3.80 ± 0.22	3.71 ± 0.22^a^
**NEFA (mmol/L)**			
Control	0.51 ± 0.03	0.45 ± 0.04^a^	0.48 ± 0.03
Aerobic	0.50 ± 0.04	0.59 ± 0.05^b^	0.47 ± 0.04
Resistance	0.49 ± 0.04	0.52 ± 0.04^ab^	0.54 ± 0.05
Combination	0.46 ± 0.03	0.49 ± 0.03^ab^	0.54 ± 0.04
**Glucose (mmol/L)**			
Control	5.35 ± 0.13	5.46 ± 0.10	5.26 ± 0.18
Aerobic	5.68 ± 0.17	5.78 ± 0.18	5.73 ± 0.10
Resistance	5.81 ± 0.46	5.81 ± 0.17	5.77 ± 0.16
Combination	5.38 ± 0.13	5.31 ± 0.10	5.55 ± 0.13
**Insulin (μUI/mL)**			
Control	14.89 ± 2.29	14.82 ± 1.66	14.76 ± 1.69
Aerobic	13.05 ± 1.01	16.67 ± 1.48*	15.87 ± 1.86
Resistance	13.98 ± 1.40	16.82 ± 1.33*	13.48 ± 1.24
Combination	14.24 ± 1.03	17.07 ± 1.33*	14.25 ± 1.25
**ApoB48 (μg/mL)**			
Control	6.75 ± 0.74	6.23 ± 0.58	7.08 ± 0.77^a^
Aerobic	5.68 ± 0.59	5.57 ± 0.78	5.58 ± 0.77^ab^
Resistance	5.92 ± 1.05	4.54 ± 0.64	4.46 ± 0.45* ^b^
Combination	5.30 ± 0.56	5.33 ± 0.82	5.04 ± 0.78^ab^
**REE (kJ/day)**			
Control	6088.4 ± 392.7		6081.9 ± 384.8
Aerobic	6388.2 ± 281.2		6283.5 ± 264.6
Resistance	6468.3 ± 312.1		6431.2 ± 302.5
Combination	6328.2 ± 319.7		6252.1 ± 291.1
**RQ**			
Control	0.84 ± 0.007		0.84 ± 0.012
Aerobic	0.84 ± 0.010		0.84 ± 0.014
Resistance	0.85 ± 0.016		0.81 ± 0.016*
Combination	0.83 ± 0.023		0.84 ± 0.017

Values are mean ± SEM (n = 64) of fasting blood measurements at baseline, week 8 and week 12. Statistical significance from baseline (within groups) is indicated by * *p* < 0.05. Statistical differences between groups at week 8 or 12 with baseline as a covariate indicated by different superscripts *p* < 0.05. TG: triglyceride. TC: total cholesterol. HDL: high density lipoprotein. LDL: low density lipoprotein. NEFA: non-esterified fatty acid. ApoB48: apolipoprotein B48. REE: resting energy expenditure. RQ: respiratory quotient.

**Figure 3:**
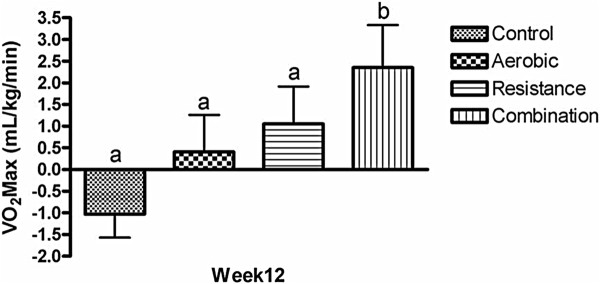
**Comparison of changes from baseline between groups for cardio-respiratory fitness.** Values are mean ± SEM (n = 64). Statistical differences between groups indicated by different superscripts, *p* < 0.05.

VO_2_max significantly increased in the Combination group compared to Control (13.3%, *p* = 0.006) (Figure 3).

## Discussion

We have previously demonstrated that a single, moderate-intensity 30-min bout of aerobic or resistance exercise improves risk factors associated with cardiovascular disease in overweight and obese adults [19], however, fasting levels of TG, cholesterol, glucose and insulin were not affected in the short-term. It is possible that some physiological changes are only seen after a longer period of training. Thus, we conducted a 12-week chronic study to explore the impact of aerobic, resistance, or combined exercise at a moderate-intensity for 30 min, five days/week. Significant decreases in body weight, BMI and total body fat were seen in the Combination group compared to Control and Resistance groups. Similarly, significant improvements were demonstrated in fat percentage, abdominal fat percentage and cardio-respiratory fitness in the Combination group compared to Control. Therefore, moderate-intensity training over 12-weeks using a variety of training modalities has beneficial effects in CVD risk factors in overweight and obese individuals compared to no exercise.

Body weight and BMI in the Combination group were significantly lower than Control and Resistance groups at 12 weeks but not Aerobic group. The absence of significant decreases in Aerobic and Resistance groups may have been due to the 30-min moderate-intensity exercise being insufficient stimuli compared to training loads reported in other studies [3,20-24]. However, Park et al. [20] also found that combination exercise was more effective for body composition improvements than aerobic exercise alone.

The Combination intervention produced the greatest improvements in body composition, significant decreases in total body fat, fat percentage, android fat percentage and gynoid fat percentage. Park et al. [20] also observed that combination exercise was more effective in decreasing visceral fat than aerobic exercise. High levels of fat, especially in the abdomen, increase the risk of developing Type 2 diabetes and CVD [25], thus combination exercise training can be beneficial in reducing this risk.

Interestingly, a similar study by Church et al. [8] observed a significant decrease in body mass in the Combination group compared to Control and Resistance groups after 9 months of training, comparable to our findings. This indicates that the effects of aerobic and resistance exercise interact to have a greater effect than either type alone. However, the mechanisms involved are unclear and further investigations are warranted.

We did not observe any significant reduction in lipids, glucose or insulin after 12 weeks of training, which is inconsistent with previous studies. Generally, those studies involved higher levels of exercise [20,26]. ApoB48 was significantly lower after training in the Resistance group compared to Control but not compared to aerobic and combination exercise. Changes to apoB48 were not reported in other studies investigating the effects of exercise training. As apoB48 is a marker for chylomicron particles, a decrease indicates a lower number of particles circulating in the blood, which is associated with decreased risk of atherosclerosis [27].

We observed a small but significant decrease in the RQ in the Resistance group. Other studies have demonstrated a decrease in resting non-protein RQ in older women after 16 weeks resistance training [28] indicating a shift in substrate utilization to increased lipid oxidation for energy. A hypocaloric diet also decreases the RQ and increases fat oxidation [29]. This matches our observation of a significant decrease in energy intake in the Aerobic and Resistance groups at week 12. This change in substrate utilization may have a beneficial impact on body fat and obesity in the long-term.

We observed a significant increase in estimated maximum oxygen uptake (VO_2max_) in the Combination group. Greater cardio-respiratory fitness has long been associated with decreased risk of disease and death [30-32]. Obese individuals with higher fitness levels generally have lower mortality rates compared to sedentary normal-weight counterparts [33]. Previous studies have shown increases in VO_2max_ from baseline levels after resistance exercise training [34] and combined aerobic and resistance exercise training [7,8,33]. In our study, a moderate-intensity of 60% of estimated HRR was used for aerobic exercise to target greater fat oxidation rather than cardio-respiratory fitness improvements per se [35]. Combination training was more effective at increasing cardio-respiratory fitness compared to Control but not aerobic and resistance training. The lack of improvement in the Aerobic group was possibly due to variability in participants. Whilst most participants displayed an increase in cardio-respiratory fitness level, some showed a reduction in cardio-respiratory fitness level (mean −5.3 mL/kg/min, n = 3) despite the training. Participant factors such as stress, anxiety and time since the last exercise session may have affected the test results. Cardio-respiratory fitness levels in the Aerobic group may eventually have increased significantly given a longer training period, such as that by Church et al., [36] which employed a six month intervention. However, our Combination intervention demonstrates that improvements in cardio-respiratory fitness in the overweight and obese can be achieved following the Australian physical activity recommendations.

The present study had a number of limitations. Participants were mainly female despite a higher prevalence of overweight and obesity in males in Australia [37]. Due to limited sample size, our study may have been underpowered to detect significant changes in some variables. As several groups of participants were staggered over a 15–month period, seasonal changes may have been a factor [38]. Reported energy intake in all groups decreased over the course of the intervention and this may have contributed to changes in body weight and composition. Monitoring the intensity and frequency of exercise was another challenge. Despite the completion of exercise diaries and regular contact with the researcher, we were reliant on participant honesty and accuracy in their self-reported information. If participants in the aerobic group did not complete the prescribed exercise this may explain the lack of improvements in the current study.

## Conclusion

A 12-week training program of resistance or combined exercise at a moderate-intensity for 30-min, five days/week resulted in unique improvements to the cardiovascular risk profile in overweight and obese participants compared to no exercise. Currently, there are no specific recommendations for the type of exercise the overweight and obese should engage in. From our observations, combination exercise gave greater benefits for weight loss, fat loss and cardio-respiratory fitness than aerobic and resistance training modalities. Therefore, combination exercise training should be recommended for overweight and obese adults in National Physical Activity Guidelines.

## Competing interests

The authors declare that they have no competing interests.

## Authors’ contributions

SH coordinated the trial, conducted data collection and had input into the manuscript. APH had input into the writing of the manuscript and SSD provided statistical oversight. SDD developed the statistical analysis protocol and performed statistical analysis in conjunction with SH. SP conceived and designed the study, supervised the study and the statistical analysis and mentored SH. All authors read and approved the final manuscript.

## Pre-publication history

The pre-publication history for this paper can be accessed here:

http://www.biomedcentral.com/1471-2458/12/704/prepub
